# Harnessing noncanonical crRNA for highly efficient genome editing

**DOI:** 10.1038/s41467-024-48012-x

**Published:** 2024-05-07

**Authors:** Guanhua Xun, Zhixin Zhu, Nilmani Singh, Jingxia Lu, Piyush K. Jain, Huimin Zhao

**Affiliations:** 1 Department of Bioengineering, University of Illinois at Urbana-Champaign, Champaign, IL 61801 USA; 2 Carl R. Woese Institute for Genomic Biology, University of Illinois at Urbana-Champaign, Champaign, IL 61801 USA; 3 Department of Chemical and Biomolecular Engineering, University of Illinois at Urbana-Champaign, Champaign, IL 61801 USA; 4 Department of Chemical Engineering, University of Florida, Gainesville, FL 32611 USA

**Keywords:** Genetic engineering, CRISPR-Cas9 genome editing, Gene expression

## Abstract

The CRISPR-Cas12a system is more advantageous than the widely used CRISPR-Cas9 system in terms of specificity and multiplexibility. However, its on-target editing efficiency is typically much lower than that of the CRISPR-Cas9 system. Here we improved its on-target editing efficiency by simply incorporating 2-aminoadenine (base Z, which alters canonical Watson-Crick base pairing) into the crRNA to increase the binding affinity between crRNA and its complementary DNA target. The resulting CRISPR-Cas12a (named zCRISPR-Cas12a thereafter) shows an on-target editing efficiency comparable to that of the CRISPR-Cas9 system but with much lower off-target effects than the CRISPR-Cas9 system in mammalian cells. In addition, zCRISPR-Cas12a can be used for precise gene knock-in and highly efficient multiplex genome editing. Overall, the zCRISPR-Cas12a system is superior to the CRISPR-Cas9 system, and our simple crRNA engineering strategy may be extended to other CRISPR-Cas family members as well as their derivatives.

## Abstract (web summary)

The inclusion of base Z has the potential to heighten the binding affinity between complementary nucleic acids. Here, the authors integrated base Z into CRISRP-Cas12a crRNA to augment the interaction between the crRNA and the target DNA, resulting in a significant enhancement of editing efficiency.

## Introduction

The clustered regularly interspaced short palindromic repeat (CRISPR) associated protein (Cas) based system has been extensively explored for genome editing and gene regulation in various organisms in the past decade^1–5^. The most widely used Cas nuclease is derived from *Streptococcus pyogenes* (SpCas9), which is also the first programmable nuclease with robust activity in eukaryotic cells^6,7^. Nonetheless, to facilitate the clinical therapeutic application of SpCas9, it is imperative to undergo multiple rounds of gRNA screening to minimize the risk of non-specific cleavage in the genome^8^. In recent years, additional Cas family members with unique advantages over Cas9 have been identified^9,10^. One notable example is AsCas12a, an RNA guided class II nuclease derived from *Acidaminococcus sp*. AsCas12a shows intrinsically higher specificity than SpCas9^11,12^, which is likely due to its lower tolerance to guide-target mismatches^13,14^. More importantly, the capacity to modify multiple genetic elements simultaneously is crucial for unraveling and regulating gene interactions and networks in complex cellular functions^15^. However, existing genome engineering tools have limitations concerning the number and types of perturbations that can be executed simultaneously. Cas12a exhibits a robust ability to carry out multiplex genome editing, whereas Cas9 lacks such capability^15^.

There is a growing interest of using Cas12a for clinical genome editing because of its intrinsic high fidelity^11,12,16–18^. Moreover, unlike the ~100-mer sgRNA of Cas9, its short 40-43-mer crRNA^19^ can be readily chemically manufactured. However, the application of the CRISPR-Cas12a system for human genome editing is still limited by its relatively low editing efficiency^19–24^. In recent years, various approaches have been developed to improve the Cas12a-based genome editing efficiency in mammalian cells, which are based on either protein engineering^24–28^ or RNA engineering^20,22,29,30^. Employing chemically modified guide RNA represents a key approach in RNA engineering. Previous studies are predominantly concentrated on chemical modifications of its sugar group^31,32^ and backbone^33,34^, whereas investigations pertaining to base modification of guide RNA remain rare. One study revealed that guide RNAs containing universal bases facilitate Cas9/Cas12a recognition of polymorphic sequences, albeit at the cost of reduced specificity^35^. Another study suggests that incorporating pyrimidine analogs in guide RNA might decrease immunogenicity, thereby potentially enhancing editing efficiency. Nonetheless, it was found that the modification on adenines in guide RNA had an adverse effect on the cleavage efficiency^36^. In addition, there were attempts to enhance the binding affinity between crRNA and the Cas12a protein^20,22^. Most recently, base Z was utilized in the guide RNA to enhance the Cas9 and Cas12a on-target efficiency^37^. In this work, the authors have only briefly explored the application of Z-RNA (Z-mRNA and Z-gRNA) and the majority of their CRISPR application studies primarily involved in vitro cleavage assays. Moreover, the authors performed limited genome editing experiments and the work lacks essential genome editing data with Cas12a. To the best of our knowledge, there is no precedent in literature where the genome editing efficiency of Cas12a is enhanced by increasing the interaction between guide RNA and its target complementary DNA strand through base modification.

**Fig. 1:**
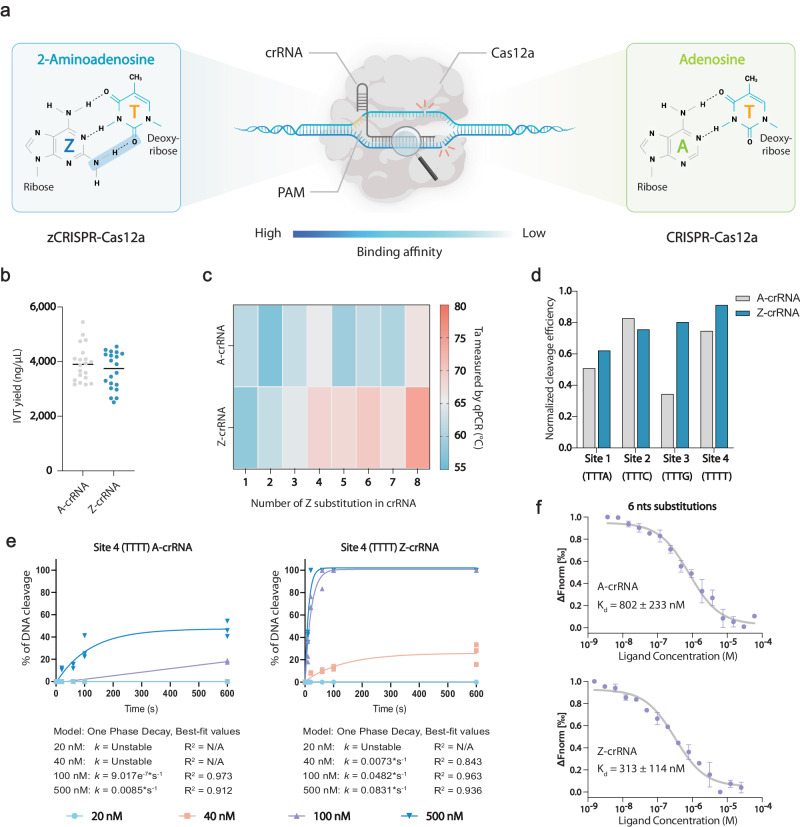
Overview of zCRISPR-Cas12a and its in vitro characterization. **a** Schematic of zCRISPR-Cas12a with enhanced binding affinity between crRNA and target DNA by the extra hydrogen bond (highlighted in blue) offered by Z:T pairing. Created with BioRender.com. **b** In vitro transcription (IVT) yield of crRNA, quantified by NanoDrop 2000/2000c Spectrophotometer. *n* = 20. **c** The heatmap of Ta value between crRNAs and their complementary ssDNAs measured by QuantStudio^TM^ 3 Real-Time PCR System. *n* = 3. **d** Normalized in vitro cleavage efficiency of A-crRNA and Z-crRNA mediated AsCas12a-based cleavage on linearized plasmid. The agarose gel electrophoresis bands were quantified by GelAnalyzer 19.1 software. **e** In vitro cleavage activity kinetics analysis. AsCas12a RNPs (both A-crRNA and Z-crRNA) were titrated into dsDNA substrates (10 nM) containing target site with TTTT PAM used in Fig. 1d. Cleavage reactions were sampled at distinct time points (10 s, 20 s, 60 s, 100 s, and 600 s), and substrate cleavage was assessed through capillary electrophoresis. Data was fitted using a one-phase decay model, and the corresponding *k* values are indicated below each figure. *n* = 3. **f** Measurement of AsCas12a RNP and dsDNA substrate interactions by Microscale Thermophoresis (MST) assays. The representative target site with six base Z substitutions in crRNA was utilized to quantify binding affinity between AsCas12a RNPs and associated dsDNA substrates. *K*_d_ values representing the dissociation constants are provided below each figure. Error bars, s.e.m.; *n* = 3.

In this work, we report a CRISPR-Cas12a crRNA engineering strategy by incorporating 2-aminoadenine (Z) into crRNA (Z-crRNA) to improve the binding affinity and target recognition, which results in a genome editing tool superior to the most widely used CRISPR-Cas9 system (Fig. 1a). Base Z alters the Watson-Crick two hydrogen bonds pairing between adenine and thymine (A:T) to three hydrogen bonds pairing (Z:T), which can increase thermal stability, sequence specificity, and nuclease resistance^38–41^. Indeed, the substitution of A with Z in crRNA dramatically improved the on-target editing efficiency of Cas12a while maintaining its intrinsic low off-target effect in mammalian cells.

## Result

### Z-crRNA exhibits increased binding affinity towards target DNA

We hypothesized that the extra hydrogen bonds from the Z:T pairs lead to higher binding affinity between crRNA and its target complementary single stranded DNA (ssDNA). To test this hypothesis, we measured the thermostability of the binary complex of in vitro synthesized crRNA (Fig. 1b, Fig. S1) and its corresponding ssDNA. Fluorescence signals from eight binary complexes (which contain incremental numbers of A or Z base in the crRNA spacer region, Fig. S2a) were collected during the annealing step^42^ (Fig. S2b). As expected, the findings indicated that the Z-crRNA has an increased propensity to form a binary complex with its complementary ssDNA at high temperatures, which was further augmented with a greater number of Z-base substitutions, i.e., Z-crRNA-ssDNA complex had a higher temperature of annealing (Ta value) than A-crRNA-ssDNA complex (Fig. 1c, Fig. S2a), suggesting that the binding affinity of Z-crRNA-ssDNA is stronger than that of the A-crRNA-ssDNA. Interestingly, the maximum raw fluorescence of Z-crRNA-ssDNA was lower than that of A-crRNA-ssDNA, which is consistent with the gel electrophoresis result (Fig. S1b) that dye molecules hardly intercalated into Z-crRNA. To explore the interaction between the AsCas12a nuclease-crRNA complex and its associated dsDNA substrate, we utilized the microscale thermophoresis (MST) assay. This enabled us to determine the dissociation constant (*K*_d_) values for eight target sites, each featuring varying A-to-Z base substitutions. The outcomes validated our hypothesis that introducing base Z within the crRNA could indeed increase the binding affinity between the crRNA and its complementary ssDNA or the ribonucleoprotein (RNP) and dsDNA substrate as the Z-crRNA exhibited a lower *K*_d_ value at most sites with the exception being the site featuring two substitutions (Fig. 1f, Fig. S3b).

### Z-crRNA facilitates Cas12a cleavage activity

To investigate whether Z-crRNA is able to mediate the cleavage by Cas12a, we performed an in vitro DNA cleavage assay using linearized plasmids and AsCas12a. Indeed, Z-crRNA can mediate the targeted DNA cleavage efficiently and outperform the A-crRNA at three out of four sites at the initial testing, particularly those with TTTT PAM (Fig. 1d, Fig. S4a). The latter is unexpected because the TTTT PAM is known to have a relatively low cleavage efficiency^24^. To thoroughly assess the efficacy of Z-crRNA in in vitro cleavage assays, we undertook a kinetics study on four sites encompassing various PAM sequences. Our observations indicated a notable enhancement in cleavage efficiency with the use of Z-crRNA, evident in both the plateau and *k* value. Z-crRNA consistently demonstrated superior performance compared to A-crRNA across all tested sites (Fig. 1e, Fig. S3a). To confirm that zCRISPR-Cas12a can increase the in vitro cleavage efficiency at sites with TTTT PAM, we selected nine additional sites with TTTT PAM for evaluation. Five of them showed improved cleavage efficiency while the rest maintained a similar cleavage efficiency as the A-crRNA (Fig. S4b). Taken together, these results (see Supplementary Text) demonstrate that Z-crRNA can be recognized by the AsCas12a nuclease for target DNA cleavage with improved efficiency.

### Z-crRNA enables enhanced Cas12a-mediated genome editing

**Fig. 2:**
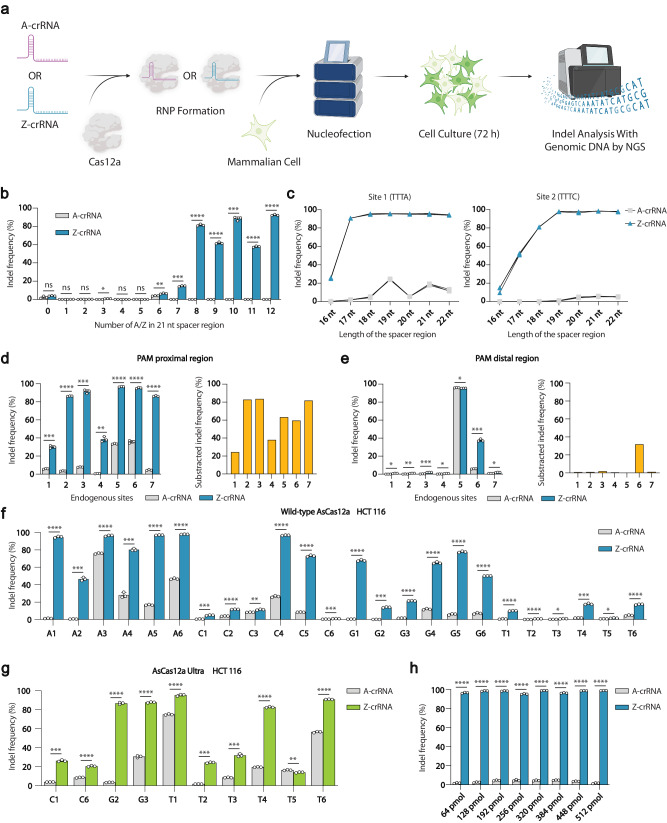
*In cellulo* characterization of zCRISPR-Cas12a. **a** Overall workflow of zCRISPR-Cas12a *in cellulo* investigation. Created with BioRender.com. **b** Genome editing performance affected by the number of Z substitution in the spacer region. The crRNAs with the incremental number of A or Z base (from 0 to 12) in the spacer region were used for genome editing. **c** Editing efficiency of A-crRNA and Z-crRNA mediated AsCas12a at two different endogenous sites with crRNA bearing variable length 3’ end truncations or extensions. **d** Characterization of the effect of Z substitution in PAM proximal region with seven endogenous sites (left), and the impact shown by the subtracted average indel frequency (the average indel frequency of Z-crRNA minus the average indel frequency of A-crRNA, right). **e** Characterization of the effect of Z substitution in PAM distal region with seven endogenous sites (left), and the impact shown by the subtracted average indel frequency (right). **f** Validation of zCRISPR-Cas12a on reported low-editing-efficiency sites. 24 previously characterized low-editing-efficiency sites^24^ with all types of PAMs were selected to verify the efficacy of zCRISPR-Cas12a in HCT116 cells. A, C, G, and T represent four kinds of PAM sequences: TTTA, TTTC, TTTG, and TTTT. **g** Performance of coupling Z-crRNA and engineered AsCas12a variant. AsCas12a Ultra was used to further improve the on-target efficiency of ten inefficient enhanced sites in (**f**). **h** Relationship between the amount of RNPs and the editing efficiency. Different amounts of RNPs were transfected into HCT116 cells. The indel frequency was measured by NGS after 72 h cell culture. Error bars, s.e.m.; *n* = 3; nt, nucleotide. Statistical analysis was performed using one-tailed Welch’s *t*-tests, ns = *p* > 0.05; * = *p* ≤ 0.05; ** = *p* ≤ 0.01; *** = *p* ≤ 0.001; **** = *p* ≤ 0.0001. Exact p-values are provided in the Source Data.

Next, we sought to investigate the Z-crRNA performance *in cellulo*. We selected eight target sites located on the *GAPDH* and *HPRT1* genes for evaluation. A-crRNA and Z-crRNA were incubated with AsCas12a nuclease to create the RNPs respectively, which were subsequently introduced into HCT116 cells through nucleofection (Fig. 2a). The T7EI assay was used to evaluate the effectiveness of on-target editing, and it was discovered that the use of Z-crRNA resulted in a significant improvement in editing efficiency, with the most improved site experiencing an increase from 17% to 65% (Fig. S5a).

Inspired by these results, we further explored the Z-crRNA design rules for achieving better performance. We hypothesized that a higher percentage of the Z base in the crRNA could lead to higher editing efficiency. Thirteen endogenous sites, targeting the *EMX1* gene with an incremental number of Z substitutions in the spacer region, were selected to investigate the on-target editing efficiency. The on-target indel frequency was measured by next generation sequencing (NGS), and the results indicate that a larger number of Z base substitutions in the spacer region resulted in better performance, which is consistent with our hypothesis (Fig. 2b). Specifically, at the selected sites of this particular gene, when the number of Z substitutions in a crRNA was increased to more than eight out of the 21 nucleotides in the spacer region, Z substitution improved the on-target editing efficiency to up to 93%, compared with nearly zero editing efficiency observed in using A-crRNA. While the utilization of Z-crRNA yielded a substantial improvement in on-target efficiency, the editing efficiency of A-crRNA in this specific gene was unexpectedly low. Furthermore, the enhanced on-target efficiency with Z-crRNA was only evident when the number of substitutions exceeded seven. To prevent any bias in target site selection, we extended our investigation to two additional genes, *TPCN2* and *RNF2*. Notably, the overall editing efficiency on these genes did not exhibit the same degree of limitation in using A-crRNA as observed in the *EMX1* gene. Instead, a consistent pattern emerged, indicating that the performance of Z-crRNA is influenced by the number of substitutions. Notably, we observed that introducing only one or two base Z substitutions in these two genes was sufficient to enhance the on-target efficiency (Fig. S5b). In addition, we investigated whether the position of the Z substitution may affect the *in cellulo* cleavage efficiency of the Cas12a nuclease, given that the PAM proximal region (also known as the seed region) plays a more critical role in target sequence recognition than the PAM distal region^43^. We selected seven sites where the Z substitutions were located in either PAM proximal or distal region from *EMX1* and *DNMT1* genes for validation (Fig. S6). On-target indel frequency was assessed at each of the seven sites, and the indel frequency resulting from A-crRNA-mediated editing was subtracted from that observed with Z-crRNA-mediated editing to show the impact of Z substitution. The results are consistent with our hypothesis that Z substitution in the PAM proximal region is more beneficial for editing efficiency improvement than in the PAM distal region (Fig. 2d, e).

### zCRISPR-Cas12a shows improved editing at low-editing-efficiency sites

Since the length of crRNA may play an essential role in target DNA recognition, we investigated the impact of the length of the spacer region on the editing efficiency. We truncated the spacer region from 22 nt to 16 nt one by one, starting at the 3’ end of the crRNA. We found that the Z substitution on crRNA efficiently promoted the on-target editing even when the spacer region was as short as 17 nt (Fig. 2c). Based on the aforementioned Z-crRNA design rules, we applied this strategy to 24 previously reported low-editing-efficiency sites^24^ and performed *in cellulo* genome editing in both HEK 293 T and HCT 116 cell lines. NGS analysis results demonstrate that the zCRISPR-Cas12a was able to elevate the editing efficiency at all characterized low-editing-efficiency sites (Fig. 2f, Fig. S5c). Notably, the largest improvement in editing efficiency relative to the canonical CRISPR-Cas12a was observed at A1 site in HCT116 cell line, which showed an increase from 1.3% to 93.9% (Fig. 2f). While statistically significant improvements were observed across all testing sites, certain sites demonstrated a more modest overall enhancement. To bolster the on-target efficiency in these specific locations, we combined our Z-crRNA strategy with a previously reported engineered variant of AsCas12a, known as AsCas12a Ultra. This approach yielded further improvements on most sites. Notably, the outcome highlighted the compatibility of our Z-crRNA with this enhanced nuclease variant, confirming its upgraded functionality (Fig. 2g, Fig. S5d). Moreover, Z-crRNA could maintain comparable editing efficiency even at RNP concentration eight times lower than what was suggested by the manufacturer (Integrated DNA Technologies, Inc.)^24^ (Fig. 2h). Taken together, these results indicate that Z-crRNA indeed significantly improved the Cas12a-based genome editing efficiency in mammalian cells.

To ensure consistent cleavage position patterns when employing Z-crRNA for on-target cleavage, we conducted in vitro cleavage assays using both A-Cas12a RNP and Z-Cas12a RNP on a dsDNA substrate. The resulting fragments were subcloned into a plasmid respectively for subsequent Sanger sequencing. Remarkably, the results demonstrated that the cleavage position pattern remained unaltered when utilizing Z-crRNA, mirroring the same pattern observed with A-crRNA (Fig. S7a, S7b). Additionally, we extended our investigation to the indel profile in a cell-based assay using Z-crRNA. Through NGS analysis, we determined that there were no notable distinctions between A-crRNA and Z-crRNA, affirming that the incorporation of Z-crRNA does not affect the indel profile in mammalian cells (Fig. S7c).

### Utilizing Z-crRNA preserves the low off-target merit of Cas12a

**Fig. 3:**
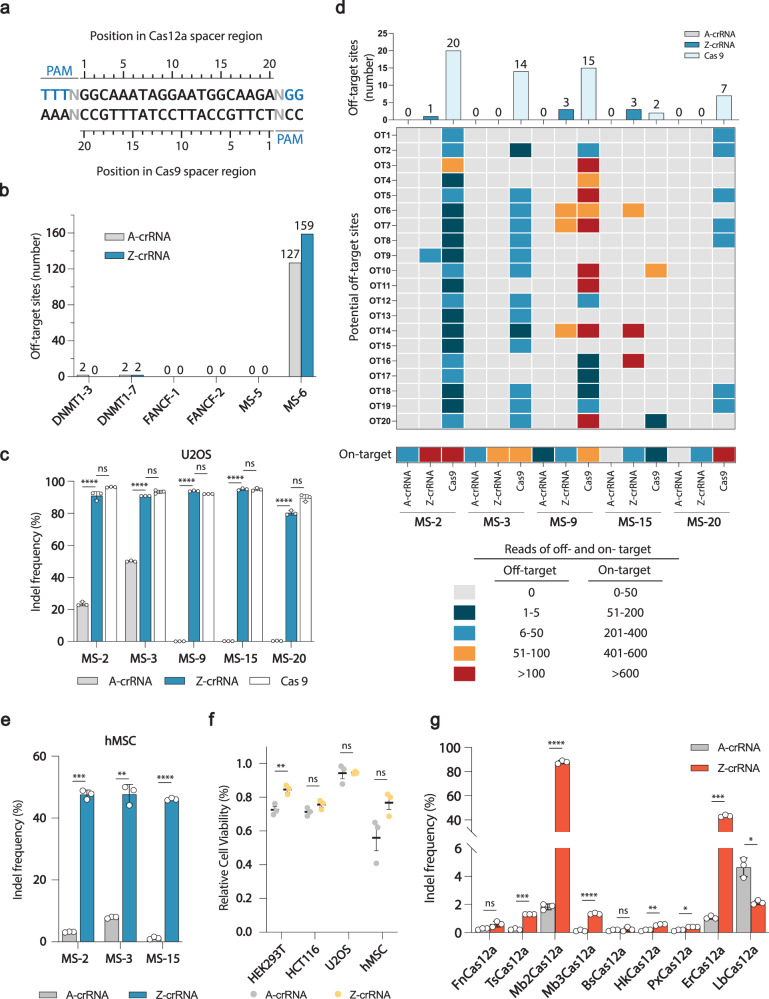
Assessment of off-target effect of CRISPR-Cas12a and zCRISPR-Cas12a and side-by-side comparison with CRISPR-Cas9. **a** Matched target sites for AsCas12a and SpCas9 that share a common protospacer sequence. **b** Summary of the total number of off-target sites detected by GUIDE-Seq in U2OS cells with six previously characterized sites^12^. **c** On-target indel frequency measured by NGS. Five low-editing-efficiency matched sites (MSs) were selected from previous study^12^ to compare the on-target editing efficiency of CRISPR-Cas12a, zCRISPR-Cas12a, and SpCas9. **d** Characteristics of off-target numbers and frequencies determined by GUIDE-Seq in U2OS cells with above five matched sites. Bar graph above represents the total numbers of off-target sites. Heatmap shows the frequencies of potential off-target sites (predicted by Cas-OFFinder, 20 off-target sites, OT1-20) detected by GUIDE-Seq and on-target sites. **e** Validation of zCRISPR-Cas12a performance in a complex cell type. Primary human Mesenchymal Stem Cells (hMSCs) were employed to validate the effectiveness, utilizing three sites matched with those in panel (**c**). **f** Cell viability assays. Cell viability assessments were conducted to evaluate the health of the four types of cells utilized in this study. The measurements were performed using the CellTiter cell viability assay. **g** Evaluation of the Z-crRNA strategy across eight Cas12a orthologs. Site 15 utilized in (**c**) was chosen to investigate the general applicability of Z-crRNA on FnCas12a, TsCas12a, Mb2Cas12a, Mb3Cas12a, BsCas12a, HkCas12a, PxCas12a, ErCas12a, and LbCas12. The aforementioned purified Cas12a proteins were incubated with A/Z-crRNA to form RNP followed by nucleofection into HEK293T cells respectively. Error bars, s.e.m.; *n* = 3. Statistical analysis was performed using one-tailed Welch’s *t*-tests, ns = *p* > 0.05; * = *p* ≤ 0.05; ** = *p* ≤ 0.01; *** = *p* ≤ 0.001; **** = *p* ≤ 0.0001. Exact *p*-values are provided in the Source Data.

To investigate whether the increased on-target editing efficiency by using Z-crRNA may sacrifice Cas12a intrinsically low off-target property, we harnessed GUIDE-Seq^12,44^ to assess the off-target cleavage frequency of the A-crRNA and Z-crRNA mediated AsCas12a systems. We selected four different previously reported target sites in *DNMT1* and *FANCF* genes as well as two matched sites^12^ as editing targets. The matched sites are the overlapping sequences in the spacer region for both Cas12a and Cas9 nucleases (Fig. 3a). The results confirmed that there was no extra off-target cleavage caused by Z substitutions in crRNA (Fig. 3b, Fig. S8), and the off-target sites detected were consistent with previously reported data^12^. Given the high specificity of the Cas12a in mammalian cells, detecting off-target cleavage events for most tested crRNAs can be challenging^45^. We employed the matched site 6, a site previously reported to have numerous off-target editing events, as our positive control to validate the quality of GUIDE-Seq. The effectiveness of the experimental performance was confirmed by the detection of a considerable number of off-target sites throughout the genome, as revealed by our results (Fig. 3b, Fig. S8).

In addition, we performed a side-by-side comparison between zCRISPR-Cas12a and CRISPR-Cas9. Five matched sites, which showed low to nearly zero editing efficiency with AsCas12a in a previous study^12^, were selected to compare both on-target editing efficiency and off-target effect of AsCas12a and SpCas9. AsCas12a-A-crRNA RNP, AsCas12a-Z-crRNA RNP, and SpCas9-sgRNA RNP were individually transfected into U2OS cells. The on-target editing efficiency and off-target effect were analyzed by NGS and GUIDE-Seq, respectively. As shown in Fig. 3c, the overall indel frequency of AsCas12a was boosted by using Z-crRNA from extremely low to up to 95%, which was comparable to that of SpCas9. Then, we compared the off-target effect between AsCas12a and SpCas9. To remove any bias resulting from site selection, we initially assessed the number of predicted off-target sites for both Cas9 and Cas12a using Cas-OFFinder. The prediction results confirmed that both Cas9 and Cas12a exhibited a comparable number of off-target sites on those five matched sites (Fig. S9f). As anticipated, the Z substitution in crRNA retained Cas12a’s low off-target effect in the genome (Fig. 3d, Fig. S9a–e). Although a few off-target sites have been identified by using Z-crRNA, they are rather limited in comparison to the off-target sites associated with Cas9. Noticeably, some identified off-target sites of Z-crRNA mediated editing from matched site 2 and 15 are the sites with the mismatches at the PAM distal region. Based on our previous data (Fig. 2c, Fig. S9a, d), we found that a 17 nt long spacer region in Z-crRNA was adequate to achieve efficient editing in the mammalian cell genome. Consequently, zCRISPR-Cas12a’s inability to differentiate such off-target sites is understandable. Moreover, the reason why A-crRNA did not result in off-target effect could be due to its intrinsically low cleavage activity with those A-crRNAs. In contrast, SpCas9 caused numerous unintended edits across the whole genome with all selected sites (Fig. 3d, Fig. S9). This result suggests that Cas9 editing is more prone to target multiple off-target sites in the genome compared to Cas12a, leading to a decrease in editing specificity. Overall, the data indicate that zCRISPR-Cas12a has the potential to significantly increase the efficiency of genome editing on target sites, and the likelihood of off-target effects may vary based on the unique sequence characteristics of the crRNA.

### Evaluation of the Z-crRNA strategy efficacy across various cell types and Cas12a orthologs

Next, we investigated the performance of zCRISPR-Cas12a in primary cells using human Mesenchymal Stem Cells (hMSCs). The three sites MS-2, MS-3, and MS-15 (refer to the side-by-side comparison experiment) were selected for the validation. As primary cells are known to be more challenging to transfect, the editing efficiency is generally not as high as observed in the U2OS cell line. Nevertheless, we found that using Z-crRNA still significantly enhanced the editing efficiency in hMSCs (Fig. 3e), and the trend observed was consistent with the efficiency in U2OS cells (Fig. 3c). Additionally, we conducted cell viability analysis on four types of cells used in this study. The results demonstrated that Z-crRNA exhibited either comparable or lower cytotoxicity when compared to A-crRNA (Fig. 3f).

Our findings with AsCas12a indicate a substantial enhancement in on-target efficiency with Z-crRNA. To evaluate the broad applicability of the Z-crRNA strategy, we investigated other Cas12a orthologs known for their mammalian cell genome editing activity^46,47^. These orthologs include *Francisella novicida* U112 (FnCas12a), *Thiomicrospira sp*. XS5 (TsCas12a), *Moraxella bovoculi* AAX08_00205 (Mb2Cas12a), *Moraxella bovoculi* AAX11_00205 (Mb3Cas12a), *Butyrivibrio sp*. NC3005 (BsCas12a), *Helcococcus kunzii* ATCC 51366 (HkCas12a), *Pseudobutyrivibrio xylanivorans* DSM 10317 (PxCas12a), *Eubacterium rectale* (ErCas12a), and *Lachnospiraceae bacterium* ND2006 (LbCas12a). In this extensive evaluation, ErCas12a and Mb2Cas12a displayed large improvements of the on-target editing efficiency when using Z-crRNA, matching the efficacy observed with AsCas12a. TsCas12a, Mb3Cas12a, HkCas12a, and PxCas12a also exhibited statistically significant improvements by applying Z-crRNA (Fig. 3g). The relatively lower editing efficiency observed with TsCas12a, HkCas12a, and BsCas12a may be attributed to their limited enzymatic activity, which has been reported in previous in vitro cleavage activity studies^48^. In the case of LbCas12a, which exhibited reduced editing efficiency with Z-crRNA, an examination of its crRNA structure revealed two consecutive “A” residues in the loop structure. Our speculation is that substituting Z in this loop may influence the secondary structure of crRNA, consequently affecting the editing efficiency. Since LbCas12a is compatible with various crRNA direct repeat sequences^48^, further investigation can be conducted using different direct repeat sequences to prevent disruption of the loop structure. To investigate the applicability of Z-crRNA to other CRISPR tools, we employed the Z-crRNA strategy with Cas9 for genome editing. Due to potential impacts on secondary structure from base Z substitutions in tracrRNA, we annealed Z-crRNA and A-tracrRNA to create a chimeric gRNA. This gRNA was then assembled with Cas9 protein for genome editing on *EMX2*, *FANCF3*, and *RUNX1* genes in HEK293T cells. The results revealed that the use of Z-sgRNA led to a decrease in Cas9 editing efficiency (Fig. S5e), consistent with the recently reported findings^37^. To gain a deeper insight into the underlying mechanism, additional structural studies should be conducted. Overall, six out of the nine tested Cas12a orthologs demonstrated enhanced on-target editing efficiency when employing Z-crRNA.

### Z-crRNA enables enhanced HDR-mediated gene integration

**Fig. 4:**
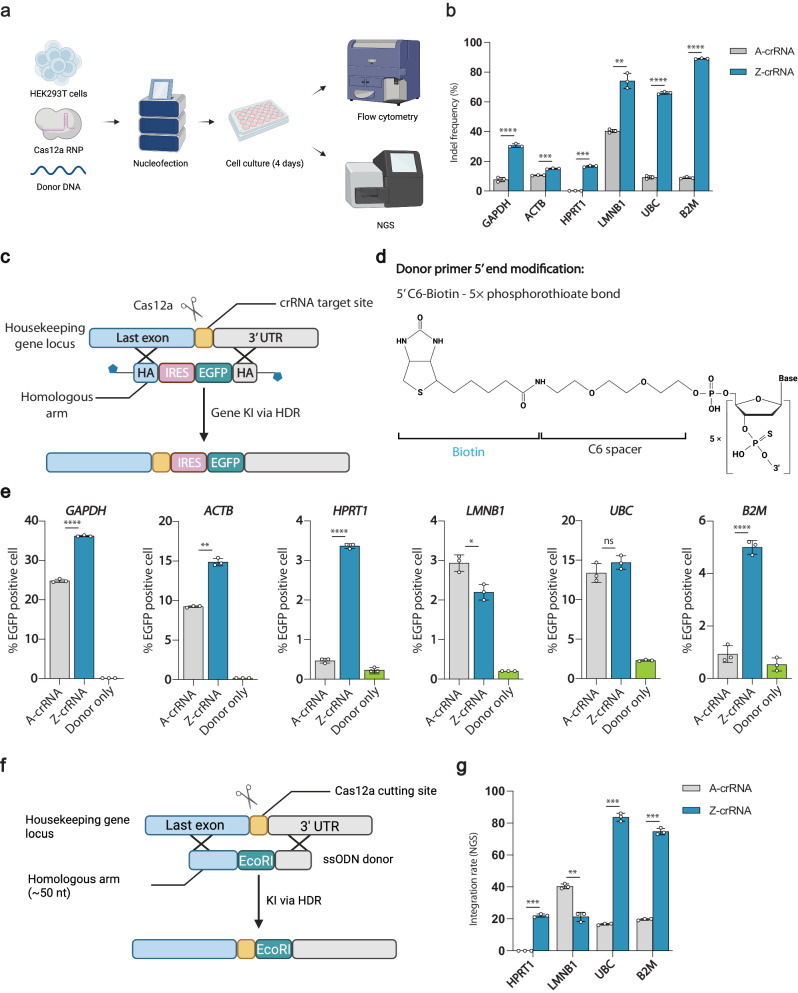
Applications of zCRISPR-Cas12a for HDR-mediated knock-in (KI). **a** Workflow for HDR-mediated knock-in (KI) experiment. HEK293T cells were transfected with CRISPR-Cas12a and zCRISPR-Cas12a RNPs along with either dsDNA (comprising IRES and EGFP) or ssODN (containing an *Eco*RI site) donor molecules. The rate of integration was assessed through flow cytometry or NGS analysis, carried out 4 days post nucleofection. **b** Analysis of indel frequency for HDR-mediated knock-in assays. On-target efficiency assessment was conducted using the same RNPs employed in the knock-in experiments. **c** Gene KI rate reporter system. A double-strand break was created in between the last exon of each housekeeping gene and its 3′ untranslated region (UTR). Gene KI was mediated by a linear dsDNA donor template that contains internal ribosomal entry site (IRES), EGFP sequence and homologous arms (HAs). Blue pentagons represent end modifications of dsDNA donors. **d** Structure of the donor end modification. The donor amplification primers were modified with 5’C6-Biotin and 5× phosphorothioate bond. **e** KI rate in HEK293T cells using modified dsDNA donors. CRISPR-Cas12a and zCRISPR-Cas12a RNPs were transfected into HEK293T cells coupled with modified donors respectively. Data were collected 4 days post nucleofection by flow cytometry. **f** ssODN-mediated KI system. A schematic of the single-stranded oligo DNA (ssODN) mediated knock-in strategy is presented. A double-strand break was induced between the last exon of each housekeeping gene and its 3’ untranslated region (UTR). Knock-in was facilitated by a ssODN donor template containing an *Eco*RI restriction site flanked by two homologous arms. **g** Integration rate measurement in ssODN-mediated KI experiment. The integration rate was evaluated through NGS analysis, with precise *Eco*RI site knock-in reads as a percentage of the total count. Error bars, s.e.m.; *n* = 3. Statistical analysis was performed using one-tailed Welch’s *t*-tests, ns = *p* > 0.05; * = *p* ≤ 0.05; ** = *p* ≤ 0.01; *** = *p* ≤ 0.001; **** = *p* ≤ 0.0001. Exact p-values are provided in the Source Data. Schematics in (**a**, **c**, **f**) were created with BioRender.com.

Since Cas12a triggers DNA cleavage at 3’ end of the spacer, the cleavage site is far away from the PAM while the original target sequence is maintained. This unique feature is beneficial to an extra opportunity to cut at the target site after indel formation, thus increasing the chance of homology-directed repair (HDR) mediated gene integration. To explore if Z-crRNA could also be harnessed to enhance HDR mediated knock-in efficiency, we used a reporter system to measure the knock-in efficiency mediated by Cas12a. The donor was designed to contain an upstream internal ribosomal entry site (IRES) and the EGFP gene flanked by 50-base-pair homology arms (HAs)^49^ (Fig. 4c). In addition, the linear donor was modified with 5’-biotinylation and phosphorothioate bonds^50^ (Fig. 4d). We separately co-electroporated A-crRNA- or Z-crRNA- Cas12a RNP with a double-stranded DNA donor into HEK 293 T cells to integrate the gene fragment between the last exon and the 3’ untranslated region (UTR) of six housekeeping genes. Knock-in efficiency was measured 4 days post nucleofection by flow cytometry, and the percentage of EGFP positive cell population was used to determine the integration rate (Fig. 4a). The HDR mediated gene knock-in efficiency was enhanced by up to 7.21-fold by using Z-crRNA for four out of six targets, including *GAPDH*, *ACTB*, *HPRT1*, and *B2M* (Fig. 4e, Fig. S10). However, the overall improvement of the integration rate of genes *HPRT1*, *LMNB1*, *UBC*, and *B2M* is limited. We speculated that the previously observed low knock-in efficiency might be attributed to variations in the expression levels of individual housekeeping genes. Therefore, we assessed the on-target efficiency across the mentioned six sites by using both CRISPR-Cas12a and zCRISPR-Cas12a. The NGS data unveiled a substantial enhancement in on-target efficiency with Z-crRNA, particularly for the sites that exhibited modest improvements in the reporter experiments (Fig. 4b). To overcome the constraints of the reporter system, we evaluated the efficiency of homology-directed repair (HDR)-mediated knock-in by utilizing single-stranded oligo DNA (ssODN) donors^24^. By integrating an *Eco*RI restriction site in the cutting site, we were able to assess the knock-in efficiency through enzyme digestion check followed by NGS analysis (Fig. 4a, f). The results revealed a significant overall enhancement in integration efficiency, with the most remarkable improvement observed at the UBC site, where the knock-in efficiency surged from 16% to 86% (Fig. 4g). Collectively, these results confirm that the zCRISPR-Cas12a can significantly improve HDR mediated knock-in efficiency compared to CRISPR-Cas12a in mammalian cells.

### The use of Z-crRNA enhanced the capability for multiplex genome editing

**Fig. 5:**
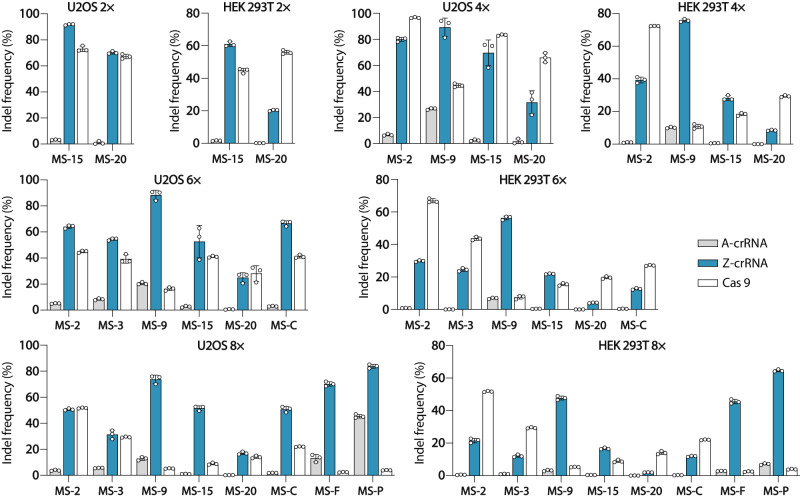
Applications of zCRISPR-Cas12a for multiplex genome editing. Analysis of the multiplex genome disruption efficiency in U2OS and HEK293T cells. Five matched sites (MSs) used in previous experiments (Fig. 3c) and other three matched sites^57^, *COL8A1* (MS-C), *FGF18* (MS-F), and *P2RX5-TAX1BP3* (MS-P), were selected for multiplex genome editing assessed by NGS. Double, quadruple, sextuple, or octuple A-crRNAs, Z-crRNAs, or sgRNAs were used to form the corresponding RNPs followed by nucleofection into cells simultaneously. 2×: double-gene knockout; 4×: quadruple-gene knockout; 6×: sextuple-gene knockout; 8×: octuple-gene. Error bars, s.e.m.; *n* = 3.

Simultaneous perturbation of multiple genes can aid in uncovering and controlling the gene interactions and networks that drive cellular functions^15^. However, current genome engineering technologies face difficulties in executing simultaneous multiplex perturbations, both in terms of the number and kind of modifications^51^. To investigate whether the Z-crRNA can facilitate multiplex genome editing in mammalian cells, we tested the multiplexing capacity of zCRISPR-Cas12a by targeting up to eight endogenous sites simultaneously. We assessed multiplex gene editing in both U2OS and HEK293T cells, employing the RNP delivery method for both Cas12a and Cas9, as the Z-crRNA cannot be encoded genetically. As shown in Fig. 5, zCRISPR-Cas12a demonstrated a significant improvement in multiplex gene editing as compared to CRISPR-Cas12a. It is noteworthy that as the number of guide RNAs increases, the Cas9 editing efficiency significantly decreases at site 15 and 20, while zCRISPR-Cas12a showed even better performance than CRISPR-Cas9 at five out of eight sites for octuple-gene editing in U2OS cell line (Fig. 5). The zCRISPR-Cas12a editing efficiency at site 9 exhibited a high level of effectiveness, with the efficiency up to 94.3%, 90.6%, and 75.9% for quadruple, sextuple, and octuple-gene editing, respectively, in U2OS cell line. In contrast, Cas9 demonstrated lower efficiency, with rates of only 46.2%, 17.5%, and 6.8% for the same conditions. For the remaining sites tested in multiplex genome editing, zCRISPR-Cas12a performed comparable to or better than Cas9. Overall, the results indicate that zCRISPR-Cas12a has a greater capability for efficient multiplex genome editing in mammalian cells, as evidenced by the superior outcomes observed.

## Discussion

In summary, our results provide an important proof-of-concept that the on-target potency of CRISPR nucleases can be augmented through enhancing the binding affinity between guide RNA and its DNA target (see Supplementary Discussion). This enhanced editing capability is attributed to the stronger binding affinity offered by Z:T three-hydrogen-bonds pairing. By incorporating the noncanonical base Z into crRNA, the resultant zCRISPR-Cas12a showcased drastically improved on-target editing efficiency without compromising its low off-target effect virtue in mammalian cells, which is more advantageous than the most widely used CRISPR-Cas9 due to its comparable on-target editing efficiency, significantly lower off-target effect, and higher multiplexing capacity. However, the heightened binding affinity between Z-crRNA and target DNA could potentially induce extra off-target edits by Cas12a in the genome. To mitigate this risk, the high-fidelity Cas12a variants can be employed to significantly reduce the likelihood of off-target occurrences^26^. As our data demonstrated the great compatibility of Z-crRNA for Cas12a variant, we believe Z-crRNA can further enhance the on-target efficiency of high-fidelity Cas12a variants while maintaining its upgraded specificity. Nevertheless, the Z-gRNA strategy was not able to enhance the editing efficiency of SpCas9, likely attributable to the intricate structure of the gRNA. Obtaining additional structural information could provide a deeper understanding of the underlying mechanism, aiding in the design of Z-crRNA for enhanced genome editing activity. We expect that our crRNA engineering strategy can be extended to other Cas proteins and CRISPR-based genome editing tools such as base editor, prime editor, and CRISPRa/i systems^52–55^.

## Methods

### Guide RNA synthesis by in vitro transcription (IVT)

For Cas12a crRNA synthesis, DNA template oligos (contain SP6 promoter sequence, Supplementary Data File 1) for IVT were ordered from IDT (Integrated DNA Technologies, Inc.). 5 μM complementary oligos were annealed in 1 × NEB buffer 3.0 (New England Biolabs, Inc.) using the following program: 98 °C 5 min, 0.1 °C/s cool down to 4 °C. HiScribe^TM^ SP6 RNA Synthesis Kit (NEB, E2070S) was used for IVT reactions according to the manufacturer’s protocol. To generate Z-incorporated crRNA, ZTP (TriLink Biotechnologies, N-1001, 2-Amino-ATP) was used to replace ATP in the reaction (Fig. S1a). Reactions were incubated at 37 °C for 10 h to reach higher concentration. For Cas9 sgRNA synthesis, Precision gRNA Synthesis Kit (Thermo Scientific, A29377) was used to create the template DNA for IVT. DNA oligos were ordered from IDT as per kit’s instruction. To remove DNA template, 25 μL of nuclease-free water with 2 μL of DNase I (NEB, M0303S) were added to each 25 μL reaction, and the reactions were incubated at 37 °C for 15 min. Synthesized RNAs were purified using RNA Clean & Concentrator-5 kit (Zymo, R1016), and the concentrations were quantified by the NanoDrop 2000/2000c (Thermo Scientific) and Qubit 4 fluorometer. RNAs were stored at −80 °C until use.

### In vitro cleavage assay

The synthesized crRNAs were diluted to 10 μM with nuclease-free water. Diluted crRNAs were pre-incubated with AsCas12a proteins (IDT, 1081069) or LbCas12a proteins (IDT, 10007922) to form the ribonucleoproteins (RNPs) in the 1 × NEB 2.0 buffer using a 1:1.2 ratio of Cas12a:crRNA. The final concentration of RNP was designed to 1 μM. After 15 min incubation at room temperature, the RNPs were mixed with 1 μL 10 × Reaction Buffer (NEB 2.0), 1 μg purified plasmid (Supplementary Data File 1), 10 U *Nde*I restriction enzyme, and added with nuclease-free water up to 10 μL. The reaction was incubated at 37 °C for 30 min. The digestion products were analyzed by running 1% agarose gel. For kinetics study, 10 nM dsDNA substrate was incubated with various amount of A/Z-AsCas12a RNPs (20 nM, 40 nM, 100 nM, and 500 nM). Cleavage reactions were sampled at distinct time points (10 s, 20 s, 60 s, 100 s, and 600 s), and substrate cleavage was assessed through capillary electrophoresis (Agilent).

### Annealing temperature (Ta) measurement

The synthesized crRNAs with various A/Z base content and their complementary ssDNAs were diluted to 10 μM. 1 μL of each crRNA and its complementary ssDNA were added in a 10 μL solution with 2 × EvaGreen (Biotium, 31000-T), 10 mM phosphate (pH 7.4), and 100 mM NaCl. QuantStudio^TM^ 3 Real-Time PCR System was used to record the raw fluorescence during the annealing step with the following program: 95 °C 2 min, cool down to 10 °C with the ramp of 0.05 °C/s, fluorescence was measured every 0.05 s (Fig. S2b). Ta values were calculated as follows:

$${{{{{\rm{crRNA}}}}}}-{{{{{\rm{ssDNA\; binary}}}}}}\,{{{{{\rm{complex}}}}}}\,{Ta}=\frac{\overline{{T}_{{A}_{\min }}}-\overline{{T}_{{A}_{\max }}}}{2}$$

where Ta represents the temperature of annealing (Ta), and T_A_ represents the raw fluorescence associated temperature.

### Microscale Thermophoresis (MST) assays

50 nt DNA oligonucleotides and their complementary strands were synthetized at the 250 nmole scale (IDT, HPLC 95% pure). For dsDNA formation, the oligonucleotides were dissolved in duplex buffer at a concentration of 100 μM. Equal volumes of complementary strands were then combined, heated to 95 °C for 5 min, and subsequently cooled to 4 °C. To remove any ssDNA, the mixture was treated with Exonuclease (New England Biolabs). Purification of the dsDNA was achieved using the Monarch® PCR & DNA Cleanup Kit (5 μg, New England Biolabs). The concentration of the dsDNA was determined using an Invitrogen™ Qubit™ 4 Fluorometer.

The binding affinity between AsCas12a and DNA was measured using Monolith NT.115 (Nanotemper Technologies). AsCas12a was fluorescently labelled using the Monolith Protein Labeling Kit RED-NHS 2nd Generation (Amine Reactive, product number MO-L011). A volume of 90 μL AsCas12a sample (10 μM) in the labelling buffer was mixed with 10 μL dye solution (300 μM) for 30 min at room temperature in the dark. Next, the AsCas12a sample was loaded to column B (Nanotemper MO-L011) and eluted with 450 μL of assay buffer (50 mM Tris-HCl, pH 7.4, 150 mM NaCl, 5 mM EDTA, 5 mg/mL BSA, 0.05% Tween-20). The labelled AsCas12a sample was incubated with crRNA at a ratio of 1:1.2 to form the AsCas12a-A-crRNA RNP or AsCas12a-Z-crRNA RNP. To perform the MST assay, we first combined 10 μL of the labeled sample with 10 μL of DNA. This DNA was present in 16 varying serial dilutions in the assay buffer, allowing the reaction to proceed for 5 min to permit binding. The samples were then loaded into Monolith NT.115 capillary (Nanotemper Technologies) and measured by using 60% RED as the excitation power and a medium MST power setting. The binding measurement was repeated three times. Data analysis was performed using Nanotemper affinity analysis software.

### Mammalian cell culture and nucleofection

HCT116 cells (ATCC, CCL-247) were cultured in McCoy’s 5 A medium supplemented with 10% FBS. HEK293T (ATCC, CRL-3216) and U2OS (ATCC, HTB-96) cells (a generous gift from Dr. Andrew Belmont at UIUC) were cultured in Advanced DMEM supplemented with 10% FBS and 1 × GlutaMax (ThermoFisher, 35050061). Primary human Mesenchymal Stem Cells (hMSC) was obtained from ATCC (PSC-500-012) and cultured in Mesenchymal Stem Cell Basal Medium (PSC-500-030) supplemented with Bone Marrow-Mesenchymal Stem Cell Growth Kit Components (PSc-500-041). All cells were cultured at 37 °C in a 5% CO_2_ incubator. To form the RNP, nuclease and crRNA (sgRNA) were diluted with PBS to a total volume of 10 μL followed by the incubation at room temperature for 10–20 min. For U2OS and HCT116 cell lines, 1 × 10^6^ cells were transfected using SE Cell Line Nucleofector Kit with RNP containing 320 pmol crRNA and 192 pmol AsCas12a Nuclease (IDT, 1081069), AsCas12a Ultra Nuclease (IDT, 10001273), or LbCas12a Nuclease (IDT, 10007923) using DN-100 program and EN-113 program respectively on a Lonza 4D-Nucleofector according to manufacturer’s instructions. 1 × 10^6^ HEK293T cells were transfected using SF Cell Line Nucleofector Kit with RNP complex containing 320 pmol crRNA and 192 pmol AsCas12a Nuclease or AsCas12a Ultra Nuclease using DS-150 program. All nucleofections were supplemented with 300 pmol Cas12a Electroporation Enhancer (IDT, 1076301). Cas12a orthologs validation was conducted on HEK293T cells with the same condition of AsCas12a mentioned above, involving FnCas12a, TsCas12a, Mb2Cas12a, Mb3Cas12a, BsCas12a, HkCas12a, PxCas12a, and ErCas12a (generous gifts from Dr. Piyush K. Jain at the University of Florida). For hMSCs, 0.5 × 10^6^ cells were transfected using P1Primary Cell Nucleofector Kit with RNP containing 320 pmol crRNA and 192 pmol AsCas12a Nuclease (IDT, 1081069) using FF-104 on a Lonza 4D-Nucleofector according to manufacturer’s instructions. For Cas9 based genome editing experiments, U2OS cells were transfected using SE Cell Line Nucleofector Kit with RNP containing 320 pmol sgRNA and 192 pmol SpCas9 Nuclease (IDT, 1081059) using DN-100 program on a Lonza 4D-Nucleofector according to manufacturer’s instructions. All nucleofections were supplemented with 300 pmol Cas9 Electroporation Enhancer (IDT, 1075916). Genomic DNA was extracted 72 hours after nucleofection using the Quick-DNA Miniprep Kit (Zymo, R1055). Genomic DNAs were stored at −20 °C until use.

### Cell viability assay

1 × 10^6^ HEK293T/HCT116/U2OS/hMSC cells were transfected with A-crRNA-RNP and Z-crRNA-RNP by nucleofection respectively. 48 h after nucleofection, culture media was carefully aspirated away and 200 ul PBS was added per well followed by equal volume of the CellTiter-Glo reagent (Promega, G7570). The reagents were mixed for 2 min on an orbital shaker to induce cell lysis and then the plate was incubated at room temperature for 10 min before measurement. Luminescence signal was detected with the SpectraMax M5 plate reader (Molecular Devices) with an integration time of 1000 ms. The absolute luminescence signal of each well was then normalized to the average signal of control group (cells without nucleofection) to obtain the relative cell viability.

### GUIDE-Seq

U2OS cell line was used for GUIDE-Seq experiments. 1 × 10^6^ cells were transfected using SE Cell Line Nucleofector Kit with RNP containing 320 pmol crRNA and 192 pmol Cas12a Nuclease (or 320 pmol sgRNA and 192 pmol Cas9 Nuclease) using DN-100 program. RNP and 100 pmol of end-protected double-stranded oligodeoxynucleotide (dsODN) containing an *Nde*I restriction site were co-transfected into U2OS cells. Genomic DNA was extracted 72 h after nucleofection using a Quick-DNA Miniprep Kit. Targeted editing sites were amplified by specific primers sets accordingly using KOD Xtreme Hot Start DNA Polymerase (Sigma-Aldrich, 71975-3). *Nde*I digestion was performed to determine the integration efficiency to ensure the GUIDE-Seq experiment is valid (integration rate >10%). 20 μL digestion reaction contains 2 μL 10 × NEB CutSmart buffer, 20 U *Nde*I, and 200 ng PCR product. The reaction was incubated at 37 °C for 1 h. Restriction-fragment length polymorphism (RFLP) assay was performed. Briefly, the cleavage fragments were run and quantified by a capillary electrophoresis instrument (Agilent Fragment Analyzer). For the creation of sequencing libraries, we followed the published protocol^56^ with minor modifications: the sheared genomic DNAs were end-repaired by using NEBNext® Ultra™ II End Repair/dA-Tailing Module (NEB, E7546S); The barcoded Y-adapter was ligated to the repaired genomic DNA by utilizing NEBNext® Ultra™ II Ligation Module (NEB, E7595S). High-throughput sequencing libraries were generated after tag-specific amplification, and the libraries were sequenced using an Illumina MiSeq sequencer. Data were analyzed using open-source software^44^. Un-demultiplex FASTQ sequence data (paired end reads along with dual-index sample barcodes) were processed using a GUIDE-Seq Python-based workflow (https://github.com/aryeelab/guideseq).

### T7EI assays

T7 endonuclease I (T7EI) mutation detection kit (IDT, 1075932) was used to determine Cas12a editing efficiencies. The targeted loci were amplified from genomic DNA using KOD Xtreme Hot Start DNA Polymerase with 20 ng of genomic DNA as template. PCR products were denatured and followed by annealing with the addition of T7EI reaction buffer as per the manufacture’s protocol. The annealed PCR products were digested with T7 endonuclease I at 37 °C for 1 hour. Fragment analyzer (Agilent) was used to estimate the modification percentages.

### Targeted deep sequencing by NGS

On-target sites were amplified from genomic DNA using KOD Xtreme Hot Start DNA Polymerase with primers containing Illumina adaptors (Supplementary Data File 1) overhang nucleotide sequences (Illumina Nextera XT Index Kit v2 Set A, FC-131-2001). KAPA HiFi HotStart ReadyMix (Roche, KK2602) was used to generate dual-indexed sequencing libraries. The libraries were sequenced by Illumina MiSeq-Nano. OutKnocker 2.0 beta (http://www.outknocker.org/outknocker2.htm) was used to analyze the indel frequency (with 2% allele threshold).

### HDR mediated reporter gene knock-in

IRES-EGFP containing dsDNA donor was amplified from the plasmid (generated in our group, Supplementary Data File 1) using PrimeSTAR Max polymerase (Takara, R045A) with homology arm-containing primers. The 50 bp homology arms are incorporated with primers listed in Supplementary Data File 1. PCR products were initially gel-purified using GeneJET gel extraction kit (Thermofisher, K0691) followed by another round of PCR to scale up the amount of the donor. Lyophilization was used to condense donors to get a concentration around 2 μg/μL. 10 μg of each donor was used in EGFP knock-in nucleofection. 1 × 10^6^ HEK293T cells were transfected using SF Cell Line Nucleofector Kit with Cas12a RNP using DS-150 program on a Lonza 4D-Nucleofector according to the manufacturer’s instruction. All nucleofections were supplemented with 300 pmol Cas12a Electroporation Enhancer, and medium was supplemented with HDR enhancer (IDT, 10007910). Flow cytometry was performed after 4 days to assess the EGFP positive cells rate. Cells were trypsinized and resuspended in ~ 500 μL PBS for analysis with BD LSRFortessa Cell Analyzer (BD Biosciences). At least 30,000 events were recorded for each sample. Data were analyzed using the BD FACSDiva software (Version 7.0). Gating strategy has been shown in Fig. S11b.

### Multiplex genome editing

Multiple crRNAs (total 320 pmol) were used in RNP formation. 1 × 10^6^ U2OS cells were transfected using SE Cell Line Nucleofector Kit with RNP containing 320 pmol crRNAs (or sgRNAs) and 192 pmol AsCas12a (or SpCas9) Nuclease using DN-100 program on a Lonza 4D-Nucleofector according to manufacturer’s instructions. 1 × 10^6^ HEK293T cells were transfected using SF Cell Line nucleofector Kit with RNP using DS-150 program. All nucleofections were supplemented with 300 pmol Cas12a (or Cas9) Electroporation Enhancer. Genomic DNA was extracted approximately 72 h after nucleofection using a Quick-DNA Miniprep Kit. Genomic DNAs were stored at −20 °C until use.

### Statistics and reproducibility

All experiments were performed at least three biologically independent experiments, the results are shown as mean s.e.m. For datasets with two groups comparisons were made between groups by one-tailed Welch’s *t*-tests. GraphPad Prism was used for plotting and graphing.

### Reporting summary

Further information on research design is available in the Nature Portfolio Reporting Summary linked to this article.

### Supplementary information

Peer Review File

Supplementary Information

Supplementary Data File 1

Reporting summary

### Source data

Source Data

## Data Availability

All data are available in the main text or the supplementary materials. All targeted amplicon sequencing data used in this study are available in the NCBI Short Read Archive with BioProject ID PRJNA1090801 [https://www.ncbi.nlm.nih.gov/bioproject/PRJNA1090801/]. Source data are provided with this paper, and extra data are available from the corresponding author upon request.

## Code availability

Codes for searching specific Z-crRNA are available at GitHub [https://github.com/danielxun25/Z-crRNA-Finder.git].
