# Supplementary material for: Harnessing noncanonical crRNA for highly efficient genome editing
Source: Nat Commun. 2024 May 7;15:3823. doi: 10.1038/s41467-024-48012-x (PMC11076584; doi:10.1038/s41467-024-48012-x)
Supplement: Supplementary file 2 — Supplementary Information [file 41467_2024_48012_MOESM2_ESM.docx]

**SUPPLEMENTARY INFORMATION**

**Harnessing Noncanonical crRNA for Highly Efficient Genome Editing**

Guanhua Xun^1,2+^, Zhixin Zhu^2,3+^, Nilmani Singh^2^, Jingxia Lu^3^, Piyush K. Jain^4^, Huimin Zhao^1,2,3*^

^1^Department of Bioengineering, University of Illinois at Urbana-Champaign, Illinois 61801, USA.

^2^Carl R. Woese Institute for Genomic Biology, University of Illinois at Urbana-Champaign, Illinois 61801, USA.

^3^Department of Chemical and Biomolecular Engineering, University of Illinois at Urbana-Champaign, Illinois 61801, USA.

^4^Department of Chemical Engineering, University of Florida, Gainesville, Florida 32611, USA.

^+^ These authors contributed equally.

*Correspondence to: [zhao5@illinois.edu](mailto:zhao5@illinois.edu)

Table of Contents

SUPPLEMENTARY TEXT 2

Characterization of the zCRISPR-Cas12a system in vitro 2

Supplementary Discussion 2

SUPPLEMENTARY FIGURES 4

References 20

# **SUPPLEMENTARY TEXT**

## **Characterization of the zCRISPR-Cas12a system *in vitro***

To synthesize the Z containing crRNA (Z-crRNA), we replaced adenosine-5’-triphosphate (ATP) with 2-adenosine-5’-triphosphate (ZTP) in the *in vitro* transcription (IVT) reaction and used SP6 RNA polymerase to incorporate Z into crRNA (Fig. S1a). Agarose gel electrophoresis showed the resultant Z-crRNA had a correct size compared to the canonical A-based crRNA (A-crRNA). However, its band intensity was much lower than that of A-crRNA despite with the same amount of RNA post purification (Fig. S1b). This is likely due to the low dye intercalation rate of Z containing crRNA.

Given that PAM sequence may affect the Cas12a cleavage efficiency, we sought to investigate the performance of zCRISPR-Cas12a on all types of PAMs. A site (site 7 in Fig. S4b) with significant improvement in Cas12a cleavage efficiency by using Z-crRNA was selected for validation. The original TTTT PAM of the target site was mutagenized to TTTA, TTTG, and TTTC PAMs on the plasmid, respectively. *In vitro* cleavage assays were applied to test the cleavage efficiency. We observed enhanced cleavage efficiency using zCRISPR-Cas12a on all four types of PAMs (Fig. S4c). We also noticed that the Cas12a cleavage mediated by A-crRNA was more efficient on TTTA, TTTG, and TTTC PAMs, which is consistent with previous report^1^.

To further investigate the applicability of zCRISPR-Cas12a system, we tested its efficacy on the endogenous gene fragment. DNA fragments were amplified from the *DNMT1* gene in the human genome and ten sites with TTTT PAM were investigated by *in vitro* cleavage assay. The results indicate that the cleavage efficiency was improved by zCRISPR-Cas12a at five sites, and the rest of the sites had a similar cleavage efficiency as CRISPR-Cas12a (Fig. S4d). Additionally, we investigated whether zCRISPR-Cas12a is generally applicable for different Cas12a isoforms, AsCas12a and LbCas12a. We applied the same *in vitro* cleavage assay on ten sites with two types of Cas12a. The result indicates that zCRISPR-Cas12a works for different Cas12a proteins (Fig. S4e).

## **Supplementary Discussion**

Although Cas12a has the potential to be a superior alternative to Cas9 due to its intrinsically higher precision and multiplexability in genome editing, its performance for on-target editing is unsatisfactory, which has hampered its broad application. To overcome this obstacle, several strategies based on protein engineering^1-4^ and crRNA engineering^5-8^ were developed to enhance its editing efficiency, but with limited success. For example, some previously reported Cas12a variants, enAsCas12a^3^ and iCas12a^9^, only showed 2-5-fold improvements in genome editing efficiency, and the enhancement of editing efficiency on some low-efficiency-sites was low. Similarly, crRNA engineering strategies also achieved limited improvement of on-target editing efficiency^5,8,10^. Moreover, most of these protein and crRNA engineering approaches are time-consuming and labor-intensive. A simple and generally applicable strategy is highly desirable.

The focus of guide RNA chemical modifications lies in altering the sugar and backbone structures. For instance, the combination of a phosphorothioate (PS) modified backbone and 2′-O-Me modifications on the terminal five nucleotides at both ends of the crRNA has been shown to enhance editing efficiency. This is believed to be achieved by reducing crRNA vulnerability to nucleolytic cleavage^11^. Moreover, the addition of modifications known to increase RNA affinity to DNA, such as 2′-fluoro (2′-F) and S-constrained ethyl (cEt), in the PAM-distal and tracrRNA-binding regions of the crRNA, respectively, further enhanced the editing efficiency^11^. In contrast, chemical modifications of the base in guide RNA are rare, and most modifications of bases in guide RNA showed limited enhancement or had an adverse impact on cleavage efficiency^12,13^. It is important to note that base Z stands out from other artificially chemical modified bases as it is a naturally occurring base.

We systematically characterized the performance of zCRISPR-Cas12a in mammalian cell genome editing and deciphered the general rules for Z-crRNA design to achieve higher on-target editing efficiency. We applied our strategy to several previously reported low-efficiency-sites for validation, the results demonstrated that the on-target editing efficiency of those sites was improved up to a hundred-fold compared to A-crRNA mediated Cas12a. Our side-by-side comparison experiment showed that zCRISPR-Cas12a achieved an on-target editing efficiency comparable to that of the CRISPR-Cas9 system but with much lower off-target in mammalian cells. Moreover, three cell lines were harnessed to validate the efficacy of zCRISPR-Cas12a in this study, all of them exhibited the consistent result that zCRISPR-Cas12a can dramatically enhance the on-target editing efficiency. Additionally, the upgraded Cas12a system can be utilized not only for editing a single site but also for achieving accurate gene knock-in and editing multiple genes simultaneously. However, there are limitations of using Z-crRNA, as base Z cannot be genetically encoded and can only be incorporated into crRNA *in vitro*. To expand the delivery options, potential approaches include co-delivering CRISPR-Cas mRNA and Z-crRNA *ex vivo* or *in vivo* using lipid nanoparticle (LNP)-based RNA delivery methods^14^ or LNP-mediated RNP delivery^15^. Given Cas12a has ability to process its own crRNA^16^, co-delivery of Cas12a mRNA and crRNA arrays into mammalian cells for multiplexed gene editing is feasible. As a follow-up study, we will test whether Cas12a could process the Z base containing crRNA arrays and validate the efficiency for multiplexed gene editing, which we believe will open up new avenues in the genome engineering field.

# **SUPPLEMENTARY FIGURES**

**Supplementary Figure 1** | **A/Z-crRNA *in vitro* synthesis. a**, Schematic of crRNA *in vitro* transcription. SP6 promoter sequence containing dsDNA was served as the template for *in vitro* transcription. SP6 RNA polymerase (HiScribe^TM^ SP6 RNA Synthesis Kit, NEB-E2070S) was used to synthesize the crRNA. Using ATP in the reaction creates A containing crRNA. ZTP (TriLink Biotechnologies, N-1001, 2-Amino-ATP) was used to replace the ATP in the reaction to generate Z containing crRNA. crRNA yield was measured by NanoDrop 2000/2000c. Created with BioRender.com. **b**, Agarose gel electrophoresis of synthesized crRNA. 500 ng of each A or Z containing crRNA was loaded in 3% agarose gel for separation.

**
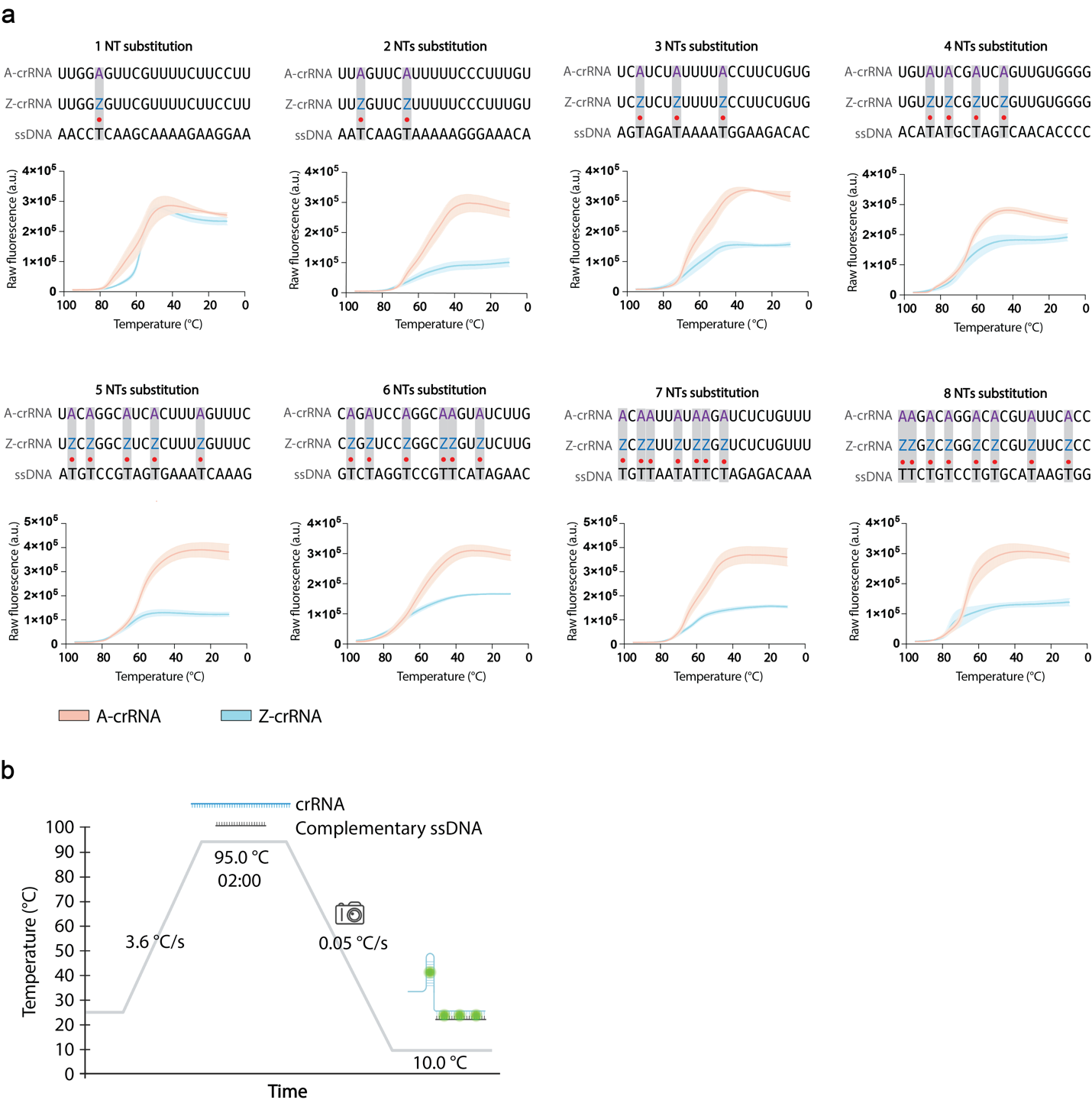
**

**Supplementary Figure 2** | **Annealing temperature (Ta) measurement. a,** Detailed sequence information of selected crRNAs with increasing number of A or Z base. crRNAs and their complementary ssDNAs were listed. The Z base substitution(s) was highlighted. The raw annealing curve was displayed below each corresponding sequence, where the red line represents the annealing temperature curve of A-crRNA/ssDNA and the blue line represents the annealing temperature curve of Z-crRNA/ssDNA. *n* = 3. **b**, Annealing temperature (Ta) measurement program. crRNA and its complementary ssDNA were mixed in the dye containing buffer. Reaction was heated up at 95 °C for 2 min to denature nucleic acids. The reaction was cooled down to 10 °C with the ramp of 0.05 °C/s to form the crRNA and ssDNA binary complex. The fluorescence was measured every 0.05 s during the cooling step. NT, nucleotide.

**Supplementary Figure 3** | **Kinetics Analysis of In Vitro Cleavage Activity and AsCas12a RNP-DNA Interaction. a,** *In vitro* cleavage activity kinetics analysis. AsCas12a RNPs (both A-crRNA and Z-crRNA) were titrated into dsDNA substrates (10 nM) containing target sites used in Fig. 1d, spanning various PAM sequences. Cleavage reactions were sampled at distinct time points (10 s, 20 s, 60 s, 100 s, and 600 s), and substrate cleavage was assessed through capillary electrophoresis. Data was fitted using a one-phase decay model, and the corresponding k values are indicated below each figure. *n* = 3. **b,** Measurement of AsCas12a RNP and dsDNA substrate interactions by Microscale Thermophoresis (MST) assays. Eight target sites with increasing substitution levels (1 nt substitution to 8 nts substitutions) were utilized to quantify binding affinity between AsCas12a RNPs and associated dsDNA substrates. K_d_ values representing the dissociation constants are provided below each figure. Error bars, s.e.m.; *n* = 3.

**Supplementary Figure 4** | **Cas12a *in vitro* cleavage assays. a,** Cleavage on initial tested four sites. Linearized plasmid (Table S3) was digested by A-crRNA and Z-crRNA mediated AsCas12a nuclease. 1% agarose gel was used to separate the cleavage products. Site 1 was TTTA PAM; Site 2 was with the TTTC PAM; Site 3 was with the TTTG PAM; Site 4 was with the TTTT PAM. **b**, Cas12a mediated *in vitro* cleavage on the sites with TTTT PAM. Other nine sites, selected from the plasmid (Table S3), were digested by A-crRNA and Z-crRNA mediated AsCas12a nuclease to verify the performance of zCRISPR-Cas12a on improving the *in vitro* cleavage efficiency. All nine sites were with the TTTT PAM at the 5’ of the protospacer region. Site 1, 2, 4, 7, and 9 showed the improved cleavage efficiency by using Z-crRNA. **c**, AsCas12a mediated *in vitro* cleavage on the same site with different PAM sequences. The original TTTT PAM for the site 7 in **b** was mutagenized to TTTA, TTTG, and TTTG PAMs on the plasmid respectively. A-crRNA and Z-crRNA were applied to mediate the AsCas12a-based cleavage on the linearized plasmid. **d**, Cas12a mediated *in vitro* cleavage on the fragment amplified from an endogenous gene. ~3.3 kb *DNMT1* gene fragment was amplified from HEK 293T cell genomic DNA. Ten target sites with TTTT PAM were selected for performing the *in vitro* cleavage assay by AsCas12a. Site 2, 3, 5, 8, and 10 showed the improved cleavage efficiency by using Z-crRNA. **e**, *In vitro* cleavage on linearized plasmid by two Cas12a isoforms. Ten target sites with TTTV PAM were chosen to verify the applicability of zCRISPR-Cas12a using different Cas12a isoforms (left: AsCas12a, right: LbCas12a). The original unprocessed gel images are available in Source Data.

**Supplementary Figure 5** | ***In cellulo* genome editing efficiency improved by using Z-crRNA.** **a**, Eight target sites with TTTN PAM were selected for assessing the performance of zCRISPR-Cas12a in *GAPDH* and *HPRT1* genes editing efficiency. Editing efficiency was estimated by T7EI assay using fragment analyzer. **b**, Genome editing performance affected by the number of Z substitution in the spacer region. The crRNAs with the incremental number of A or Z base (from 0 to 12) in the spacer region were used for *RNF2* and *TPCN2* genes editing. **c**, Validation of zCRISPR-Cas12a on reported low-editing-efficiency sites. 24 previously characterized low-editing-efficiency sites with all types of PAMs were selected to verify the efficacy of zCRISPR-Cas12a in HEK293T cells. A, C, G, and T represent four kinds of PAM sequences: TTTA, TTTC, TTTG, and TTTT. **d**, Performance of coupling Z-crRNA and engineered AsCas12a variant. AsCas12a Ultra was used to further improve the on-target efficiency of ten inefficient enhanced sites in **c**. **e**, Genome editing capabilities of Cas9 using both A-crRNA/A-tracrRNA (A-gRNA) guide RNA and Z-crRNA/A-tracrRNA (Z-gRNA) chimeric guide RNA. The validation of genome editing was conducted on the *EMX2*, *FANCF3*, and *RUNX1* genes in HEK293T cells. *n*=3. Statistical analysis was performed using one-tailed Welch’s *t*-tests, ns = *p* > 0.05; * = *p* ≤ 0.05; ** = *p* ≤ 0.01; *** = *p* ≤ 0.001; **** = *p* ≤ 0.0001. Exact p-values are provided in the Source Data.

**Supplementary Figure 6** | **Detailed crRNA sequence information of the selected targeting sites for investigating the essential part of substitution.** The seven targeting sites were selected to have the substitutions in the PAM proximal region, and the other seven sites were with the substitution in the PAM distal region. The substituted nucleotides were highlighted.

**Supplementary Figure 7** | **Investigation of Cleavage Position Pattern and Indel Profile Using Z-crRNA. a,** Exploration of cleavage position pattern. The workflow employed to study the cleavage position pattern is presented. Initially, dsDNA (1 kb) was subjected to digestion by CRISPR-Cas12a and zCRISPR-Cas12a, respectively. The resultant cleavage fragments underwent gel purification, followed by sticky end extension through DNA polymerase. Subsequently, the end-repaired fragments were separately subcloned into a plasmid. Sanger sequencing was conducted to analyze the cleavage position patterns. Created with BioRender.com. **b,** Analysis of indel profiles. Indel profile assessment is described. Genomic DNA edited by CRISPR-Cas12a and zCRISPR-Cas12a at target sites was subjected to Next-Generation Sequencing (NGS) analysis. Visualization of the indel profiles was facilitated through the utilization of the OutKnocker indel analyzing tool.

**Supplementary Figure 8** | **Off-target effect of the CRISPR-Cas12a and zCRISPR-Cas12a with six target sites.** The off-target effect was determined using GUIDE-Seq in U2OS cells. Mismatched positions in the target sites of off-targets are highlighted in color, and GUIDE-Seq read counts shown to the right of the on- and off-target sequences represent a measure of cleavage efficiency at a given site. The intrinsic poor sequence specific site, Matched Site 6, was chosen as the positive control to ensure our GUIDE-Seq experiments performed successfully. All off-target sites tested by GUIDE-Seq are consistent with the previous work^17^.

**Supplementary Figure 9** | **Off-target frequency comparison of CRISPR-Cas12a, zCRISPR-Cas12a and SpCas9 at five matched sites.** **a-e**, The off-target effect was determined using GUIDE-Seq in U2OS cells. Mismatched positions in the target sites of off-targets are highlighted in color, and GUIDE-Seq read counts shown to the right of the on- and off-target sequences represent a measure of cleavage efficiency at a given site. **f**, Off-target sites predicted by Cas-OFFinder. All matched sites’ potential off-target sites were evaluated by Cas-OFFinder software (<http://www.rgenome.net/cas-offinder/>), where we selected up to five mismatches in the spacer region. Homo sapiens (GRCh38/hg38)-Human was chosen as the target genome.

**Supplementary Figure 10** | **Representative flow cytometry analysis of the reporter gene (EGFP) knock-in efficiency.** The EGFP^+^ population (P3) in each group was analyzed using a flow cytometry gating strategy. Donor only groups were included as a control to show the background fluorescence from the donor. In these experiments, all crRNAs were with 18 nt spacer (*n*=3).

**Supplementary Figure 11** | **Representative flow cytometry analysis of the reporter gene (EGFP) knock-in efficiency and gating strategy. a**, EGFP donor knock-in efficiency measured by flow cytometry (*n* = 3). **b**, The EGFP^+^ population (P3) in each group was analyzed using a flow cytometry gating strategy. In these experiments, all crRNAs were with 20 nt spacer (*n* = 3). **c**, Gating strategy. 1) FSC-A/SSC-A was used to remove debris; 2) FSC-A/FSC-W was used to define single cells; 3) FSC-A/FITC-A was used to determine fluorescence.

# **References**

1 Zhang, L. *et al.* AsCas12a ultra nuclease facilitates the rapid generation of therapeutic cell medicines. *Nat Commun* **12**, 3908, doi:10.1038/s41467-021-24017-8 (2021).

2 Jones, S. K., Jr. *et al.* Massively parallel kinetic profiling of natural and engineered CRISPR nucleases. *Nat Biotechnol* **39**, 84-93, doi:10.1038/s41587-020-0646-5 (2021).

3 Kleinstiver, B. P. *et al.* Engineered CRISPR-Cas12a variants with increased activities and improved targeting ranges for gene, epigenetic and base editing. *Nat Biotechnol* **37**, 276-282, doi:10.1038/s41587-018-0011-0 (2019).

4 Liu, P. *et al.* Enhanced Cas12a editing in mammalian cells and zebrafish. *Nucleic Acids Res* **47**, 4169-4180, doi:10.1093/nar/gkz184 (2019).

5 Bin Moon, S. *et al.* Highly efficient genome editing by CRISPR-Cpf1 using CRISPR RNA with a uridinylate-rich 3'-overhang. *Nat Commun* **9**, 3651, doi:10.1038/s41467-018-06129-w (2018).

6 Kocak, D. D. *et al.* Increasing the specificity of CRISPR systems with engineered RNA secondary structures. *Nat Biotechnol* **37**, 657-666, doi:10.1038/s41587-019-0095-1 (2019).

7 Li, B. *et al.* Engineering CRISPR-Cpf1 crRNAs and mRNAs to maximize genome editing efficiency. *Nat Biomed Eng* **1**, doi:10.1038/s41551-017-0066 (2017).

8 Ling, X. *et al.* Improving the efficiency of CRISPR-Cas12a-based genome editing with site-specific covalent Cas12a-crRNA conjugates. *Mol Cell* **81**, 4747-4756 e4747, doi:10.1016/j.molcel.2021.09.021 (2021).

9 Ma, E. *et al.* Improved genome editing by an engineered CRISPR-Cas12a. *Nucleic Acids Res* **50**, 12689-12701, doi:10.1093/nar/gkac1192 (2022).

10 Kim, H. *et al.* Highly specific chimeric DNA-RNA-guided genome editing with enhanced CRISPR-Cas12a system. *Mol Ther Nucleic Acids* **28**, 353-362, doi:10.1016/j.omtn.2022.03.021 (2022).

11 Allen, D., Rosenberg, M. & Hendel, A. Using Synthetically Engineered Guide RNAs to Enhance CRISPR Genome Editing Systems in Mammalian Cells. *Front Genome Ed* **2**, 617910, doi:10.3389/fgeed.2020.617910 (2020).

12 Krysler, A. R., Cromwell, C. R., Tu, T., Jovel, J. & Hubbard, B. P. Guide RNAs containing universal bases enable Cas9/Cas12a recognition of polymorphic sequences. *Nat Commun* **13**, 1617, doi:10.1038/s41467-022-29202-x (2022).

13 Yang, H. *et al.* CRISPR-Cas9 recognition of enzymatically synthesized base-modified nucleic acids. *Nucleic Acids Res* **51**, 1501-1511, doi:10.1093/nar/gkac1147 (2023).

14 Gillmore, J. D. *et al.* CRISPR-Cas9 In Vivo Gene Editing for Transthyretin Amyloidosis. *N Engl J Med* **385**, 493-502, doi:10.1056/NEJMoa2107454 (2021).

15 Mirjalili Mohanna, S. Z. *et al.* LNP-mediated delivery of CRISPR RNP for wide-spread in vivo genome editing in mouse cornea. *J Control Release* **350**, 401-413, doi:10.1016/j.jconrel.2022.08.042 (2022).

16 Port, F., Starostecka, M. & Boutros, M. Multiplexed conditional genome editing with Cas12a in Drosophila. *Proc Natl Acad Sci U S A* **117**, 22890-22899, doi:10.1073/pnas.2004655117 (2020).

17 Kleinstiver, B. P. *et al.* Genome-wide specificities of CRISPR-Cas Cpf1 nucleases in human cells. *Nat Biotechnol* **34**, 869-874, doi:10.1038/nbt.3620 (2016).
