# Supplementary material for: Harnessing noncanonical crRNA for highly efficient genome editing
Source: Nat Commun. 2024 May 7;15:3823. doi: 10.1038/s41467-024-48012-x (PMC11076584; doi:10.1038/s41467-024-48012-x)
Supplement: Supplementary file 4 — Reporting summary [file 41467_2024_48012_MOESM4_ESM.pdf]

nature portfolio

Corresponding author(s): Huimin Zhao

Last updated by author(s): Mar 22, 2024

Reporting Summary

Nature Portfolio wishes to improve the reproducibility of the work that we publish. This form provides structure for consistency and transparency in reporting. For further information on Nature Portfolio policies, see our Editorial Policies and the Editorial Policy Checklist.

Statistics

For all statistical analyses, confirm that the following items are present in the figure legend, table legend, main text, or Methods section.

n/a	Confirmed	
☐	☒	The exact sample size (n) for each experimental group/condition, given as a discrete number and unit of measurement
☐	☒	A statement on whether measurements were taken from distinct samples or whether the same sample was measured repeatedly
☐	☒	The statistical test(s) used AND whether they are one- or two-sided<br>*Only common tests should be described solely by name; describe more complex techniques in the Methods section.*
☒	☐	A description of all covariates tested
☒	☐	A description of any assumptions or corrections, such as tests of normality and adjustment for multiple comparisons
☐	☒	A full description of the statistical parameters including central tendency (e.g. means) or other basic estimates (e.g. regression coefficient) AND variation (e.g. standard deviation) or associated estimates of uncertainty (e.g. confidence intervals)
☐	☒	For null hypothesis testing, the test statistic (e.g. F, t, r) with confidence intervals, effect sizes, degrees of freedom and P value noted<br>*Give P values as exact values whenever suitable.*
☒	☐	For Bayesian analysis, information on the choice of priors and Markov chain Monte Carlo settings
☒	☐	For hierarchical and complex designs, identification of the appropriate level for tests and full reporting of outcomes
☒	☐	Estimates of effect sizes (e.g. Cohen's d, Pearson's r), indicating how they were calculated

Our web collection on statistics for biologists contains articles on many of the points above.

Software and code

Policy information about availability of computer code

Data collection	Cas-OFFinder (http://www.rgenome.net/cas-offinder/) was used to predict the off-target sites for helping target site selection. Image Lab (version 5.2.1) was used for acquisition of gel images.
Data analysis	The agarose gel electrophoresis bands on gel images were quantified by GelAnalyzer (version 19.1). OutKnocker 2.0 beta (http://www.outknocker.org/outknocker2.htm) was used to analyze the indel frequency. The GUIDE-Seq workflow was run by using Python v2.7.13 to generate relevant summary outputs. Flow cytometry data were analyzed by using BD FACSDiva software (version 7.0). GraphPad Prism (version 9.5.0) was utilized for statistic analysis and data plotting.

For manuscripts utilizing custom algorithms or software that are central to the research but not yet described in published literature, software must be made available to editors and reviewers. We strongly encourage code deposition in a community repository (e.g. GitHub). See the Nature Portfolio guidelines for submitting code & software for further information.

Data

Policy information about availability of data

All manuscripts must include a data availability statement. This statement should provide the following information, where applicable:

- Accession codes, unique identifiers, or web links for publicly available datasets
- A description of any restrictions on data availability
- For clinical datasets or third party data, please ensure that the statement adheres to our policy

We declare that all data supporting the findings of this study are presented in the main text, extended data, and supplementary information. Source data are provided with this paper. Accession codes for sequencing data will be available before publication, all other raw data files available on request.

Human research participants

Policy information about studies involving human research participants and Sex and Gender in Research.

Reporting on sex and gender	N/A
Population characteristics	N/A
Recruitment	N/A
Ethics oversight	N/A

Note that full information on the approval of the study protocol must also be provided in the manuscript.

Field-specific reporting

Please select the one below that is the best fit for your research. If you are not sure, read the appropriate sections before making your selection.

☒ Life sciences ☐ Behavioural & social sciences ☐ Ecological, evolutionary & environmental sciences

For a reference copy of the document with all sections, see nature.com/documents/nr-reporting-summary-flat.pdf

Life sciences study design

All studies must disclose on these points even when the disclosure is negative.

Sample size	No sample-size calculations were performed before experiments. Three biological replicates were used for all cell-based assays. This size has previously been shown as sufficiently powered to determine statistical differences in mean values of our investigated parameters.
Data exclusions	Some preliminary data are not included for clarity and conciseness.
Replication	In vivo optimization for the genome editing performance affected by the number of Z substitution in the spacer region was only explored on one gene. In vivo optimization for the relationship between the amount of RNPs and the editing efficiency was only investigated on one gene. For the rest of experiments in this study, the target sites were selected more than two for validation. Three biological replicates were investigated for all cell-based assays such as deep-sequencing, T7EI assay, and flow cytometry.
Randomization	No randomization was performed in our study as appropriate control samples were used in our some experiments.
Blinding	Not performed and not relevant to our study.

Reporting for specific materials, systems and methods

We require information from authors about some types of materials, experimental systems and methods used in many studies. Here, indicate whether each material, system or method listed is relevant to your study. If you are not sure if a list item applies to your research, read the appropriate section before selecting a response.

Materials & experimental systems

n/a	Involved in the study
☒	☐ Antibodies
☐	☒ Eukaryotic cell lines
☒	☐ Palaeontology and archaeology
☒	☐ Animals and other organisms
☒	☐ Clinical data
☒	☐ Dual use research of concern

Methods

n/a	Involved in the study
☒	☐ ChIP-seq
☐	☒ Flow cytometry
☒	☐ MRI-based neuroimaging

Eukaryotic cell lines

Policy information about cell lines and Sex and Gender in Research

Cell line source(s)	HEK 293T cell line (ATCC, CRL-3216), , HCT 116 cell line (ATCC, CCL-247), U2OS (ATCC, HTB-96) cell line and hMSC (ATCC, PSC-500-012) cells were used in this study.
Authentication	Cells were authenticated by the supplier using STR analysis.
Mycoplasma contamination	All cell lines tested negative for mycoplasma.
Commonly misidentified lines (See ICLAC register)	None of the cell lines used in this study are listed in the ICLAC database.

Flow Cytometry

Plots

Confirm that:

☒ The axis labels state the marker and fluorochrome used (e.g. CD4-FITC).

☒ The axis scales are clearly visible. Include numbers along axes only for bottom left plot of group (a 'group' is an analysis of identical markers).

☒ All plots are contour plots with outliers or pseudocolor plots.

☒ A numerical value for number of cells or percentage (with statistics) is provided.

Methodology

Sample preparation	HEK 293T cells were trypsinized, quenched in medium, pelleted, resuspended in PBS, and filtered through a cell strainer prior to flow cytometry.
Instrument	BD LSRFortessa Cell Analyzer (BD Biosciences)
Software	BD FACSDiva software (Version 7.0)
Cell population abundance	Analytical flow had >30,000 cells.
Gating strategy	1) FSC-A/SSC-A was used to remove debris; 2) FSC-A/FSC-W was used to define single cells; 3) FSC-A/FITC-A was used to determine fluorescence. Gating for EGFP-positive (transfected) cells was based on EGFP-negative (untransfected, wild-type) cells. we have included example gating strategies in Supplementary Figure 2.

☒ Tick this box to confirm that a figure exemplifying the gating strategy is provided in the Supplementary Information.
