# Supplementary material for: Harnessing noncanonical crRNA for highly efficient genome editing
Source: Nat Commun. 2024 May 7;15:3823. doi: 10.1038/s41467-024-48012-x (PMC11076584; doi:10.1038/s41467-024-48012-x)
Supplement: Supplementary file 1 — Peer Review File [file 41467_2024_48012_MOESM1_ESM.pdf]

nature portfolio

Peer Review File

Harnessing Noncanonical crRNA for Highly Efficient Genome Editing

Open Access This file is licensed under a Creative Commons Attribution 4.0 International License, which permits use, sharing, adaptation, distribution and reproduction in any medium or format, as long as you give appropriate credit to the original author(s) and the source, provide a link to the Creative Commons license, and indicate if changes were made. In the cases where the authors are anonymous, such as is the case for the reports of anonymous peer reviewers, author attribution should be to 'Anonymous Referee' followed by a clear attribution to the source work. The images or other third party material in this file are included in the article's Creative Commons license, unless indicated otherwise in a credit line to the material. If material is not included in the article's Creative Commons license and your intended use is not permitted by statutory regulation or exceeds the permitted use, you will need to obtain permission directly from the copyright holder. To view a copy of this license, visit http://creativecommons.org/licenses/by/4.0/.

Editorial Note: This manuscript has been previously reviewed at another journal that is not operating a transparent peer review scheme. The manuscript was considered suitable for publication without further review at *Nature Communications.*
